# Supplementary material for: Splice-Junction-Based Mapping of Alternative Isoforms in the Human Proteome
Source: Cell Rep. Author manuscript; Available in PMC 2020 Jan 15. (PMC6961840; doi:10.1016/j.celrep.2019.11.026)
Supplement: 2 [file NIHMS1546469-supplement-2.zip › DF1/PXD010154/Pancreas/Pancreas_7_CPSF1_NLMVYMYLPEGECFLHPLCLGRGWASSDPP.pdf]

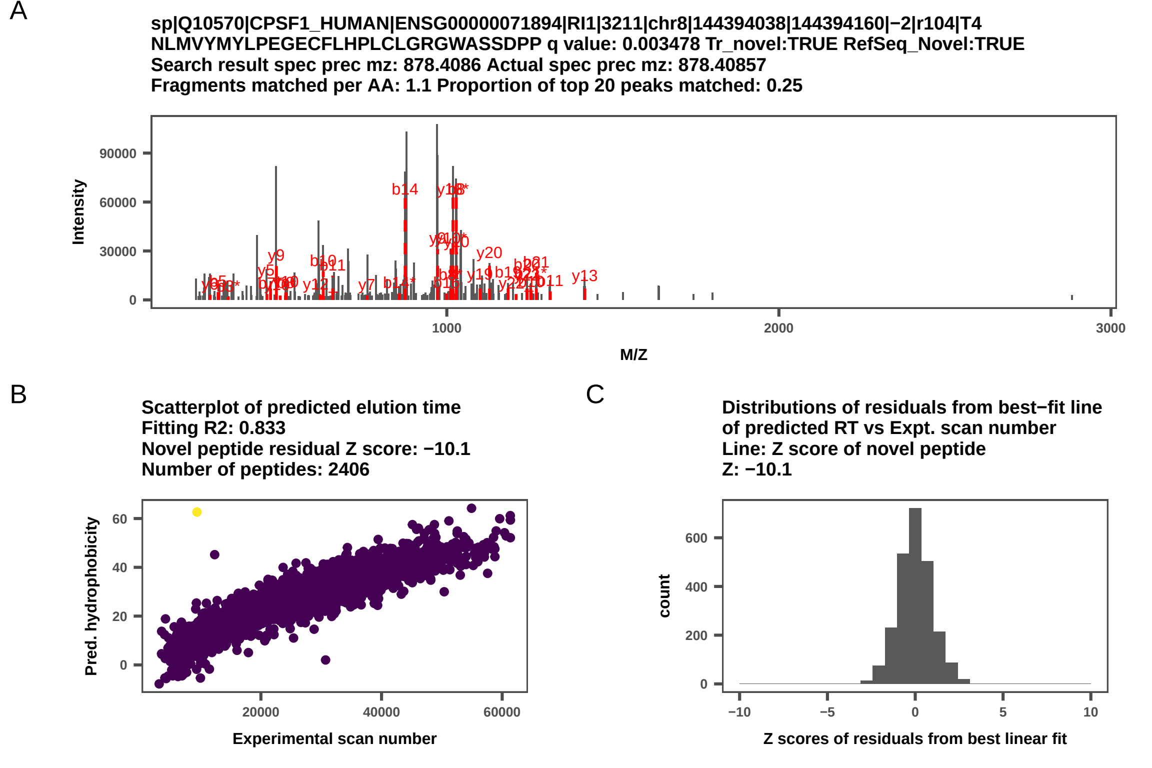
A
sp|Q10570|CPSF1_HUMAN|ENSG00000071894|RI1|3211|chr8|144394038|144394160|−2|r104|T4
NLMVYMYLPEGECFLHPLCLGRGWASSDPP q value: 0.003478 Tr_novel:TRUE RefSeq_Novel:TRUE
Search result spec prec mz: 878.4086 Actual spec prec mz: 878.40857
Fragments matched per AA: 1.1 Proportion of top 20 peaks matched: 0.25
Intensity
90000
60000
30000
0
b14
y9
y5
b10
b11
y12
y7
b14*
y20
y19
y13
1000
2000
3000
M/Z
B
Scatterplot of predicted elution time
Fitting R2: 0.833
Novel peptide residual Z score: −10.1
Number of peptides: 2406
Pred. hydrophobicity
60
40
20
0
20000
40000
60000
Experimental scan number
C
Distributions of residuals from best−fit line
of predicted RT vs Expt. scan number
Line: Z score of novel peptide
Z: −10.1
count
600
400
200
0
−10
−5
0
5
10
Z scores of residuals from best linear fit
